# RAB10 Interacts with ABCB4 and Regulates Its Intracellular Traffic

**DOI:** 10.3390/ijms22137087

**Published:** 2021-06-30

**Authors:** Amel Ben Saad, Virginie Vauthier, Martine Lapalus, Elodie Mareux, Evangéline Bennana, Anne-Marie Durand-Schneider, Alix Bruneau, Jean-Louis Delaunay, Emmanuel Gonzales, Chantal Housset, Tounsia Aït-Slimane, François Guillonneau, Emmanuel Jacquemin, Thomas Falguières

**Affiliations:** 1 Inserm, Université Paris-Saclay, Physiopathogénèse et traitement des maladies du foie, UMR_S 1193, Hepatinov, 91400 Orsay, France; amel.ben-saad@inserm.fr (A.B.S.); martine.lapalus@universite-paris-saclay.fr (M.L.); elodie.mareux@universite-paris-saclay.fr (E.M.); emmanuel.gonzales@aphp.fr (E.G.); emmanuel.jacquemin@aphp.fr (E.J.); 2 Inserm, Sorbonne Université, Centre de Recherche Saint-Antoine (CRSA), UMR_S 938, Institute of Cardiometabolism and Nutrition (ICAN), 75012 Paris, France; vauthier.v@gmail.com (V.V.); anne-marie.durand-schneider@inserm.fr (A.-M.D.-S.); alix.bruneau@charite.de (A.B.); jean-louis.delaunay@sorbonne-universite.fr (J.-L.D.); chantal.housset@inserm.fr (C.H.); tounsia.ait_slimane@sorbonne-universite.fr (T.A.-S.); 3 Université de Paris, Institut Cochin, Inserm U1016, CNRS UMR 8104, 75014 Paris, France; 4 3P5-Proteom’IC platform, Université de Paris, Institut Cochin, Inserm U1016, CNRS UMR8104, 75014 Paris, France; at.evangeline@gmail.com (E.B.); francois.guillonneau@u-paris.fr (F.G.); 5 Department of Hepatology & Gastroenterology, Charité Universitätsmedizin Berlin, 13353 Berlin, Germany; 6 Assistance Publique—Hôpitaux de Paris, Paediatric Hepatology & Paediatric Liver Transplant Department, Reference Center for Rare Paediatric Liver Diseases, FILFOIE, ERN Rare-Liver, Faculté de Médecine Paris-Saclay, CHU Bicêtre, 94270 Le Kremlin-Bicêtre, France; 7 Assistance Publique—Hôpitaux de Paris, Hôpital Saint-Antoine, Reference Center for Inflammatory Biliary Diseases and Autoimmune Hepatitis, FILFOIE, ERN Rare-Liver, 75012 Paris, France

**Keywords:** bile secretion, intracellular traffic, MDR3, phosphatidylcholine, RAB GTPase

## Abstract

ABCB4 (ATP-binding cassette subfamily B member 4) is an ABC transporter expressed at the canalicular membrane of hepatocytes where it ensures phosphatidylcholine secretion into bile. Genetic variations of ABCB4 are associated with several rare cholestatic diseases. The available treatments are not efficient for a significant proportion of patients with ABCB4-related diseases and liver transplantation is often required. The development of novel therapies requires a deep understanding of the molecular mechanisms regulating ABCB4 expression, intracellular traffic, and function. Using an immunoprecipitation approach combined with mass spectrometry analyses, we have identified the small GTPase RAB10 as a novel molecular partner of ABCB4. Our results indicate that the overexpression of wild type RAB10 or its dominant-active mutant significantly increases the amount of ABCB4 at the plasma membrane expression and its phosphatidylcholine floppase function. Contrariwise, RAB10 silencing induces the intracellular retention of ABCB4 and then indirectly diminishes its secretory function. Taken together, our findings suggest that RAB10 regulates the plasma membrane targeting of ABCB4 and consequently its capacity to mediate phosphatidylcholine secretion.

## 1. Introduction

ABCB4 (ATP-binding cassette subfamily B member 4), also named MDR3 (MultiDrug Resistance 3), is one of the main biliary transporters [1]. It is expressed at the canalicular membrane of hepatocytes where it mediates phosphatidylcholine (PC) secretion into bile [2]. In the aqueous environment of bile, PC plays a critical role in cholesterol solubilization as well as bile acid neutralization [3]. Variations in the *ABCB4* gene are associated with several rare cholestatic diseases, including Progressive Familial Intrahepatic Cholestasis type 3 (PFIC3), Low Phospholipid-Associated Cholelithiasis (LPAC) syndrome, and Intrahepatic Cholestasis of Pregnancy (ICP) [4,5]. While treatment with ursodeoxycholic acid remains efficient for the majority of patients with milder forms of ABCB4-related diseases, this is not the case for patients with PFIC3, the most severe form of these diseases, who most often require liver transplantation [6,7], stressing the unmet need for new therapeutic options. In the frame of personalized medicine, new targeted pharmacotherapies for ABCB4-related diseases have been proposed from in vitro studies and include the potentiator Ivacaftor/VX-770 as well as structural analogues of roscovitine [8,9]. In addition, AAV8- or mRNA-mediated gene therapy constitute an interesting alternative to rescue ABCB4 deficiency, as recently described in mouse models by several research groups [10,11,12,13]. In order to better characterize and understand the mode of action of new therapies, a better understanding of the molecular mechanisms regulating ABCB4 traffic and function is required.

Over the last decade, proteomic studies have become a powerful tool to decipher molecular mechanisms at the subcellular level and to provide new insights into protein biology. In the last years, a limited number of ABCB4 interacting partners were identified by yeast two-hybrid screens, including HS1-Associated protein X-1 (HAX1), the motor protein Myosin II regulatory Light Chain 2 (MLC2), Receptor for Activated C-kinase 1 (RACK1), and ERM-Binding Phosphoprotein of 50 kDa (EBP50) [14,15,16,17,18]. Aiming at identifying novel molecular partners of ABCB4, we used an immunoprecipitation approach combined with HPLC-coupled tandem mass spectrometry analyses (usually defined as “Affinity Purification-Mass Spectrometry” or AP-MS). This allowed us to identify the small GTPase RAB10 as a potential ABCB4 interactor. RAB10 belongs to the Ras-related in brain (Rab) family of proteins, which are well known as master regulators of intracellular traffic and sorting processes [19]. RAB proteins are implicated in the majority of vesicular transport steps, including vesicle formation, motility, and tethering, as well as their fusion with target membranes [19]. To date, more than 60 RAB proteins have been identified in mammals. These soluble proteins are ubiquitously expressed and can be membrane-associated with many subcellular compartments thanks to their post-translational geranyl-geranylation [20]. RAB10 was first cloned from Madin-Darby Canine Kidney cells [21]. It is mainly involved in protein trafficking from the Golgi apparatus to the plasma membrane [22]. Its implication in GLUT4 traffic as well as TLR4 exocytosis is well documented [23,24]. RAB10 also plays a key role in ciliogenesis, neuronal development, and basolateral recycling [22].

In the present study, we used biochemical and morphological approaches in cell models, namely HEK and HeLa cells. These cells allow high transfection rate and high expression level of transgenes, and they are validated models for investigating the cell biology of ABC transporters [25,26]. We found that the overexpression of RAB10-wild type (WT) or its constitutively active mutant increases ABCB4 membrane expression and function, whereas RAB10 silencing attenuates ABCB4 cell surface expression, as well as its PC secretion function. Taken together, our results indicate that RAB10 is an important regulator of ABCB4 traffic, by promoting its transport from the Golgi apparatus to the plasma membrane.

## 2. Results

### 2.1. Identification of RAB10 as a New Molecular Partner of ABCB4

**Figure 1:**
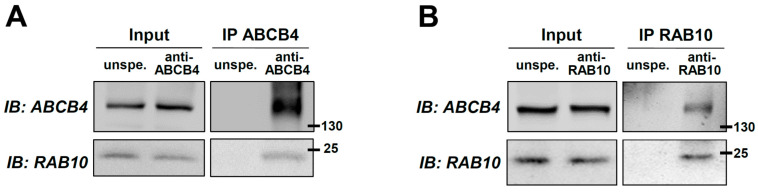
Co-immunoprecipitation of ABCB4 and RAB10 in primary human hepatocytes. (**A**,**B**) ABCB4 (A) or RAB10 (B) were immunoprecipitated from primary human hepatocyte lysates using specific antibodies. Controls were performed using unspecific antibodies (unspe.). After SDS-PAGE, the presence of ABCB4 and RAB10 in the lysates (Input) and the immunoprecipitates (IP) was detected by immunoblot (IB) as indicated. Molecular weight markers (in kDa) are indicated. These panels are representative of at least three independent experiments per condition. The presented data were cropped from full immunoblots shown in Supplementary Figure S2.

Despite the important role played by ABCB4 in bile secretion, little is known about the molecular mechanisms regulating its expression, intracellular traffic, and function. To clarify some of these mechanisms, the aim of this study was to identify key players implicated in ABCB4 regulation. For this task, an AP-MS approach was used (see details in the Section 4). Among several ABCB4 candidate interactors (Supplementary Table S1), we found the small GTPases RAB10 and RAB13 of particular interest, as RAB11 has been involved in the traffic of the canalicular bile salt export pump ABCB11 [27]. Our preliminary results indicated less encouraging results for RAB13 (data not shown), so we decided to further investigate the role of RAB10 in the intracellular traffic and function of ABCB4. To confirm the interaction between ABCB4 and RAB10, co-immunoprecipitation experiments were performed in primary human hepatocytes. RAB10 was detected after ABCB4-specific immunoprecipitation but not in the control condition with unspecific antibodies (Figure 1A). Conversely, ABCB4 was specifically detected from RAB10-immunoprecipitated complexes (Figure 1B). The same results were observed in HEK cells co-transfected with ABCB4 and RAB10-GFP (Supplementary Figure S1). These results confirm the interaction between ABCB4 and RAB10.

### 2.2. Overexpression of RAB10-WT or Its Dominant-Active Mutant Increases ABCB4 Plasma Membrane Expression and Function

**Figure 2:**
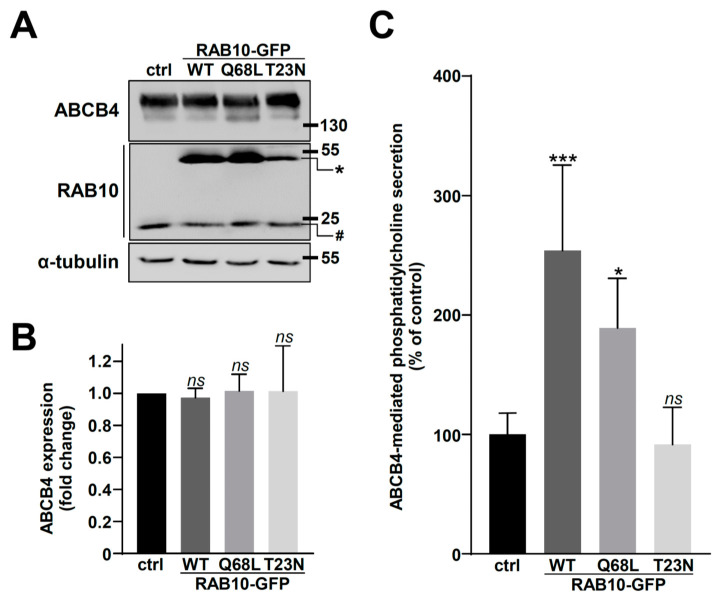
Analysis of ABCB4 expression and function after RAB10 overexpression in HEK cells. (**A**) ABCB4 and the indicated forms of RAB10-GFP were transiently expressed in HEK cells. Empty EGFP-C1 plasmid was used as control (ctrl). Forty-eight hours post-transfection, cell lysates were prepared and analyzed by immunoblot using the indicated antibodies. Molecular weight markers (in kDa) are indicated, as well as endogenous (#) and GFP-tagged (*) RAB10. This panel is representative of three independent experiments. The presented data were cropped from full immunoblots shown in Supplementary Figure S4. (**B**) Densitometry analysis of (A). The amount of ABCB4 was quantified, normalized to the amount of tubulin, and then expressed as fold change compared to the control condition (ctrl). Means (±SD) of three independent experiments are shown; ns: not significant. (**C**) HEK cells expressing both ABCB4 and the indicated forms of RAB10-GFP were used to measure ABCB4-mediated PC secretion. PC secretion was represented as a percentage of the activity for control cells (ctrl, expressing GFP alone) after background subtraction. Means (±SD) of three independent experiments performed in triplicate for each tested condition are shown; * *p* < 0.05; *** *p* < 0.005; ns: not significant.

To investigate the functional role of RAB10 in ABCB4 regulation, we first studied the effect of RAB10 overexpression on ABCB4 expression. HEK cells, co-expressing ABCB4 and different forms of RAB10-GFP (WT or its constitutively active Q68L and inactive T23N forms), or GFP alone (as control), were used. RAB10 (endogenous and GFP-tagged) and ABCB4 expression was detected by immunoblot (Figure 2A). The densitometry analyses indicated that the transient expression of the different forms of RAB10 had no effect on total ABCB4 expression (Figure 2B). However, when we studied ABCB4 function, we observed an important increase of ABCB4-mediated PC secretion in HEK cells expressing RAB10-WT or the constitutively active RAB10-Q68L, but not in cells expressing the inactive RAB10-T23N form (Figure 2C). In the absence of ABCB4-WT expression, no significant increase of basal PC efflux was observed when RAB10-WT alone was expressed (Supplementary Figure S3).

**Figure 3:**
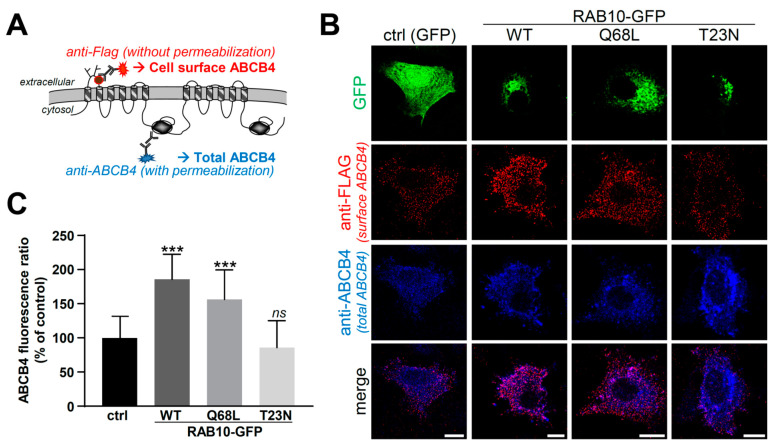
Analysis of ABCB4 localization at the plasma membrane after RAB10 overexpression in HeLa cells. (**A**) Schematic representation of the indirect immunofluorescence approach allowing the discrimination of cell surface (red) vs. total (blue) ABCB4 using anti-FLAG antibodies before permeabilization and anti-ABCB4 antibodies after permeabilization, respectively. (**B**) ABCB4-FLAG and the indicated RAB10 forms were expressed in HeLa cells. After 48 h of expression, the cell surface and total ABCB4 were labeled as schematized in (A). After immunolabeling, cell surface ABCB4 (red), total ABCB4 (blue) and RAB10-GFP (green; GFP alone for control) were visualized by confocal microscopy. This panel is representative of three independent experiments. Bars: 10 µm. (**C**) Quantification of (B). Fluorescence ratio of cell surface ABCB4/total ABCB4 was determined and represented as a percentage of the control condition (GFP alone-expressing cells). For each condition, at least 90 independent cells from three independent experiments were analyzed. Means (±SD) are represented; *** *p* < 0.005; ns: not significant.

Considering the key role played by RAB proteins in intracellular protein trafficking, we explored the hypothesis that the increased ABCB4-mediated PC secretion in cells overexpressing RAB10-WT or -Q68L might be correlated with an increase of ABCB4 expression at the cell surface. To verify this hypothesis, in a first attempt, we used polarized HepG2 cells to quantify amounts of ABCB4 present at bile canaliculi (Supplementary Figure S5). However, this did not allow an appropriate quantification of fluorescence intensities at the canalicular membrane, due to the important clustering of ABCB4-associated fluorescence in the very small area of bile canaliculus. Alternatively, we studied ABCB4 immunolocalization in HeLa cells co-expressing a modified version of ABCB4 with a FLAG tag in its first extracellular loop (Figure 3A) and the different forms of RAB10-GFP as above. We used HeLa cells for this approach since they are much more spread than HEK cells and thus allow better quantification analyses. In the absence of cell permeabilization and using anti-FLAG antibodies, this strategy allows for exclusively detecting cell surface ABCB4 (Figure 3A,B, red), while the use of anti-ABCB4 antibodies after cell permeabilization reveals the ABCB4 total population (Figure 3A,B, blue). The specificity of the plasma membrane staining of ABCB4 using anti-FLAG antibodies is supported by the observation that the intracellularly retained ABCB4-I541F variant [28] with the same FLAG tag (ABCB4-I541F-FLAG) does not display this specific signal, while this is partially rescued upon treatment with cyclosporin A (Supplementary Figure S6), known to rescue the plasma membrane targeting of this variant [29]. In line with our hypothesis and in agreement with results regarding ABCB4 function (Figure 2B), we observed that the amount of ABCB4 at the cell surface was markedly increased in cells expressing RAB10-WT or -Q68L in comparison to the control condition with GFP alone (Figure 3B). To confirm these observations, we quantified the fluorescence ratio of cell surface/total ABCB4 in the different experimental conditions and we observed a significant increase of this fluorescence ratio when RAB10-WT or its dominant-active form were expressed (Figure 3C). It is important to note that the reference for these quantifications of fluorescence intensities was “total ABCB4”, a parameter that is not influenced by the subcellular localization of ABCB4. Altogether, these results suggest that RAB10 is involved in the plasma membrane targeting of ABCB4, an obligatory process allowing its function of PC secretion towards the extracellular environment.

### 2.3. RAB10 Silencing Reduces ABCB4 Plasma Membrane Expression and Decreases Its Function

**Figure 4:**
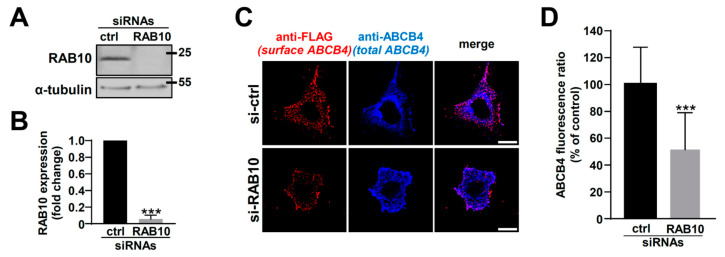
Effect of RAB10 knockdown on ABCB4 localization in HeLa cells. (**A**) Forty-eight hours after siRNA transfection in HeLa cells (Control, ctrl or anti-RAB10), RAB10 expression was analyzed by immunoblot, as in Figure 2A. This panel is representative of three independent experiments. The presented data were cropped from full immunoblots shown in Supplementary Figure S7. (**B**) Densitometry analysis of (A), as performed in Figure 2B. Means (±SD) of at least three independent experiments are shown; *** *p* < 0.005. (**C**) HeLa cells treated as in (A) were used to analyze ABCB4-FLAG localization at the plasma membrane, as performed in Figure 3B. This panel is representative of three independent experiments. Bars: 10 µm. (**D**) Quantification of (C), as performed in Figure 3C. Means (±SD) are represented; *** *p* < 0.005.

Then, we investigated the effect of RAB10 silencing on ABCB4 cell surface expression using a siRNA approach. The transfection of specific anti-RAB10 siRNAs in HeLa cells induced an important knockdown of endogenous RAB10 (Figure 4A). The quantification of these experiments indicated that RAB10 expression was decreased by more than 90% in comparison with cells transfected with control siRNAs (Figure 4B). In this RAB10 knockdown condition, using the specific plasma membrane labeling described above, we observed that less ABCB4 was localized at the plasma membrane than in the control condition (Figure 4C), which was confirmed by the quantification of these experiments (Figure 4D).

**Figure 5:**
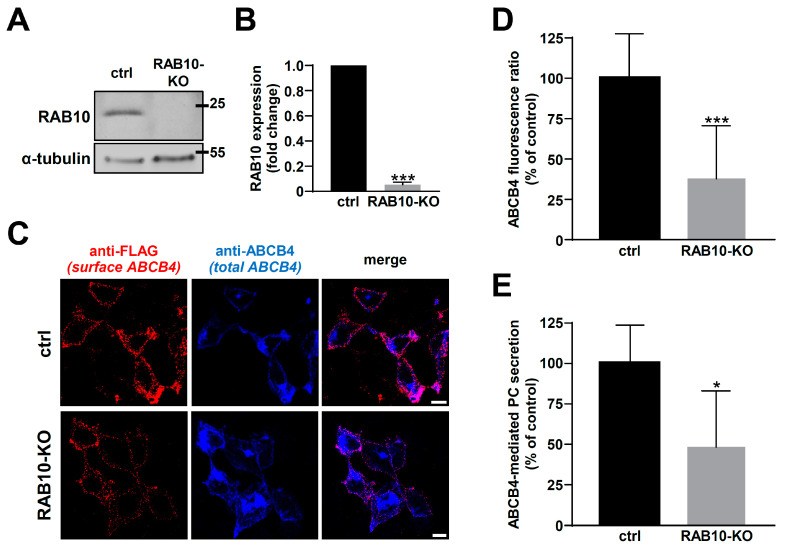
Effect of RAB10 knockdown on ABCB4 localization and function in HEK cells. (**A**,**B**) RAB10 expression in control (ctrl) and RAB10-KO HEK cells was analyzed (A) and quantified (B) as in Figure 4A,B. (A) is representative of three independent experiments and (B) represents the means (±SD) of three independent experiments; *** *p* < 0.005. The presented data were cropped from full immunoblots shown in Supplementary Figure S7. (**C**) HEK cells treated as in (A) were used to analyze ABCB4-FLAG localization at the plasma membrane, as performed in Figure 3B. This panel is representative of three independent experiments. Bars: 10 µm. (**D**) Quantification of (C), as performed in Figure 3C. Means (±SD) are represented; *** *p* < 0.005. (**E**) Control (ctrl) and RAB10-KO HEK cells were used to measure ABCB4-mediated PC secretion, as performed in Figure 2C. Means (±SD) of three independent experiments are shown; * *p* < 0.05.

To confirm the effect of RAB10 silencing on ABCB4 cell surface expression, we used RAB10-KO HEK cells generated by the CRISPR/Cas9 approach. This strategy was complementary to siRNA transfection and less aggressive for the cells in the frame of assays aiming at measuring ABCB4-mediated PC secretion. Immunoblot analyses of RAB10 expression indicated an important knockdown of protein expression in HEK cells (Figure 5A) to more than 90% compared to control cells (Figure 5B). Then, ABCB4 cell surface expression was determined as aforementioned. In line with our previous results in HeLa cells, an important decrease in ABCB4 cell surface expression was detected in RAB10-KO HEK cells, in comparison to control cells (Figure 5C; quantification in Figure 5D). We also examined the effect of RAB10 silencing by CRISPR/Cas9 on ABCB4 function in HEK cells: ABCB4-mediated PC secretion was strongly impaired in RAB10-KO cells in comparison to control cells (Figure 5E). Altogether, these results indicate that RAB10 knockdown diminishes plasma membrane expression levels of ABCB4, and thus indirectly its PC secretion function towards the extracellular environment.

### 2.4. RAB10 Promotes ABCB4 Trafficking from the Golgi Apparatus to the Plasma Membrane

**Figure 6:**
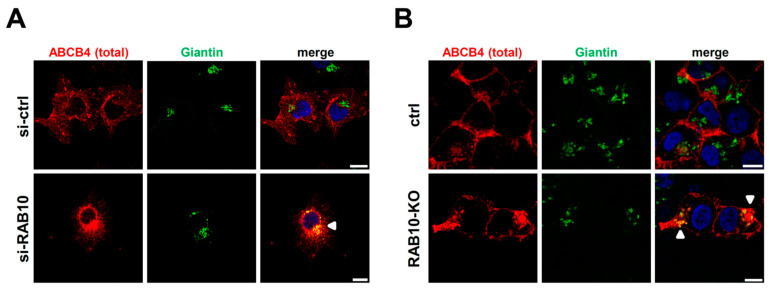
Effect of RAB10 depletion on total ABCB4 subcellular distribution. (**A**,**B**) HeLa cells transfected with control (si-ctrl) or anti-RAB10 (si-RAB10) (A), and control (ctrl) vs. RAB10-KO HEK cells (B) were used to analyze ABCB4 localization by indirect immunofluorescence and confocal microscopy. ABCB4 (red) and giantin (green) were labeled using specific antibodies while nuclei were stained with Hoechst 33,342 (blue). Each panel is representative of at least three independent experiments per condition. White arrows indicate colocalization of ABCB4 with giantin at the Golgi apparatus. Bars: 10 µm.

Since we observed a considerable reduction of ABCB4 expression at the cell surface after RAB10 knockdown, both in HeLa and HEK cells (see Figure 4 and Figure 5) without significant modification of total ABCB4 expression (Supplementary Figure S8), we inferred that RAB10 knockdown caused an intracellular retention of ABCB4. As previously reported [22], we found that RAB10 was preferentially associated with the Golgi apparatus, colocalizing with giantin (Supplementary Figure S9). We thus hypothesized that the plasma membrane targeting of ABCB4 might be impaired at post-Golgi steps when RAB10 is knocked down. The total ABCB4 distribution was examined by confocal microscopy in both HeLa and HEK cells. Under control conditions, ABCB4 displayed a significant plasma membrane staining (Figure 6A,B, upper panels). However, after RAB10 knockdown, ABCB4 was less present at the plasma membrane with an increased colocalization with the Golgi marker giantin, both in HeLa cells (Figure 6A, lower panels) and HEK cells (Figure 6B, lower panels). These results provide evidence that RAB10 is involved in ABCB4 trafficking from the Golgi apparatus to the plasma membrane.

## 3. Discussion

ABCB4 plays an important role in bile secretion [1,5]. However, little is known regarding its molecular regulation. In the present study, to further understand ABCB4 biology, we explored ABCB4 interactome. Using an AP-MS screen, we identified the small GTPase RAB10 as a novel ABCB4 binding partner. However, using this method, we did not identify the few other known partners of ABCB4 [18]. This could be explained by: (i) a weak sensitivity of the technique; (ii) the stringent conditions of the immunoprecipitation; or (iii) the poor overlap of the different techniques and studies, as already reported for the analysis of ABCB11 interactome [30]. RAB10 belongs to the RAB protein family, known as key players in intracellular traffic processes [19]. Interestingly, it has already been reported that several RAB proteins interact with and regulate the intracellular traffic of several ABC transporters. Indeed, RAB11 colocalizes with both ABCB11 and ABCC2 and regulates their recycling to the canalicular membrane [27,31]. RAB4 and RAB5 were also reported as ABCB1 regulators [32,33]; and RAB5a, RAB7, RAB4, RAB11a, and RAB27a are implicated in the regulation of ABCC7/CFTR traffic and function [34]. More recently, RAB10 has also been shown to be implicated in CFTR targeting to the plasma membrane [35].

In the present study, we show that the transient overexpression of RAB10-WT or its dominant-active mutant (RAB10-Q68L) significantly increases ABCB4 expression at the cell surface and consequently its ability to secrete PC outside the cells. In contrast, the overexpression of the dominant-inactive form RAB10-T23N had no significant effect on ABCB4 localization or function, except for a tendency to decrease ABCB4 expression at the cell surface and its function. These results might be explained by the fact that this constitutively inactive form is less expressed than the other RAB10 forms (see Figure 2A), which might be due to its reduced stability, as previously reported [36]. This hypothesis is strengthened by the fact that RAB10 depletion, by both siRNA or CRISPR/Cas9 approaches, reduces ABCB4 cell surface expression and function as well as it induces its intracellular accumulation in the Golgi apparatus. Since RAB10 has been implicated in post-Golgi trafficking of GLUT4 and TLR4 [23,24], we can speculate that this RAB protein is also necessary for vesicular trafficking of ABCB4 from the Golgi apparatus to the plasma membrane. The fact that we only observed a partial decrease of ABCB4 cell surface expression and function in RAB10-knocked down and RAB10-KO cells (see Figure 4 and Figure 5) might be due to the redundancy of RAB10 function with other RAB proteins. Indeed, functional similarity and redundancy between RAB8, RAB10, and RAB13 have been reported [22]. Further investigation will be required for a deeper analysis of the role of RAB protein redundancy on ABCB4 intracellular traffic and function. It is also interesting to note that ABCB4 staining at the plasma membrane appears as punctuated (ABCB4-FLAG staining in Figure 3B and Figure 4C), suggesting its partition in specialized microdomains at the plasma membrane. Whether the transporter is localized in “raft-like” structures or not, the latest having already been suggested [37], would require further investigation.

ABCB4 defects are associated with several cholestatic liver diseases [2,4]. Interestingly, during the last years, many cholestatic like-phenotypes were associated with mutations in genes encoding traffic regulators, such as the motor protein MYO5B, the vacuole protein sorting-associated protein VPS33B, and its interacting protein VIPAS39 [38,39,40,41]. This highlights and supports the importance of correct ABCB4 trafficking to ensure its function and thereby a normal bile flow [41]. Thus, it seems important to more deeply explore molecular mechanisms regulating ABCB4 intracellular traffic. Moreover, based on the present study, it is tempting to speculate that *RAB10* mutations or malfunction could lead to diseases mimicking ABCB4 deficiency, arguing for the research of *RAB10* mutations in patients with unexplained cholestatic diseases. In this respect, it is of interest that RAB10 dysregulation was reported in some cases of hepatocellular carcinoma, which may support its key role in protein trafficking in hepatocytes [42].

In conclusion, using cell models, we report here that RAB10 is a novel ABCB4 molecular partner and is a key regulator of its intracellular traffic from the Golgi apparatus to the plasma membrane. Further work will be necessary to determine the physiological relevance of this interaction and its role in polarized hepatocytes. Finally, we expect that an improved understanding of ABCB4 regulation will help the development of new therapeutic options for patients with cholestatic diseases related to ABCB4 defects.

## 4. Materials and Methods

### 4.1. Plasmids, Cell Culture and Transfection

Two different constructs encoding human ABCB4 were used: pcDNA3-ABCB4, which was previously described [28], and pcDNA3-ABCB4-FLAG, a modified version of ABCB4 with a FLAG tag (DYKDDDDK) within its first extracellular loop (between Ser 99 and Leu 100), prepared by Genscript (Piscataway, NJ, USA). Constructs encoding human RAB10-WT (pEGFP-RAB10-WT) and its constitutively active (pEGFP-RAB10-Q68L) and inactive (pEGFP-RAB10-T23N) forms, all with N-terminal GFP, were a kind gift from Mark McNiven (Department of Biochemistry and Molecular Biology, Mayo Clinic, Rochester, MN, USA), which were prepared as described [43].

Primary human hepatocytes were prepared as described [44] and kindly provided by the Human HepCell platform (ICAN, Paris, France). Human embryonic kidney cells (HEK-293, herein referred to as HEK; ATCC^®^-CRL-1573TM) and HeLa cells (ATTC^®^-CCL-2TM) were grown at 37 °C with 5% CO_2_ as described [45]. HEK and HeLa cells were transfected using Turbofect (Thermo Fisher Scientific, Villebon-sur-Yvette, France) at a ratio of reagent:DNA of 2:1 according to the manufacturer’s instructions. JetPrime (PolyPlus Transfection, Illkirch, France) was used for small interfering RNA (siRNA) transfection according to the manufacturer’s instructions.

### 4.2. RNA Interference and CRISPR/Cas9 System

For RAB10 silencing experiments, anti-RAB10 siRNA (ON TARGETplus human RAB10 siRNA SMARTpool) and scramble control siRNA (ON TARGETplus non-targeting control pool) from Horizon Discovery (Cambridge, UK) were used. RAB10-knockout (KO) HEK cells were established using the CRISPR/Cas9 technology as previously described [46]. Briefly, sgRNA guide sequences targeting human RAB10 were designed using the CRISPR design tool from Horizon Discovery (sense, 5′–GCGTACGTCTTCTTCGCCATT–3′ and antisense, 5′–AATGGCGAAGAAGACGTACGC–3′) and cloned into pSpCas9(BB)-2A-Puro plasmid (PX459, Addgene). After sequence verification, the resulting plasmid was transfected into HEK cells, and 24 h later, 2 µg/mL puromycin (Gibco) was added to the culture medium for the selection of transfected cells.

### 4.3. Immunoprecipitation and Mass Spectrometry Analyses (AP-MS)

For this approach, ABCB4 was immunoprecipitated from primary human hepatocytes and ABCB4-expressing HEK cells, then potential interactors were identified by tandem mass spectrometry. Primary human hepatocytes (2 × 10^7^ cells) and HEK cells expressing ABCB4 (10^7^ cells) were lysed in lysis buffer (20 mM Tris-HCl pH 8.0, 137 mM NaCl, 2 mM EDTA, 1% Triton X-100) containing protease inhibitors (cOmplete™ Protease Inhibitor Cocktail, Sigma, Saint-Quentin-Fallavier, France). The lysates were centrifuged at 14,000 rpm, 10 min at 4 °C, then supernatants were collected. After supernatant preclearing 1 h at 4 °C with 40 µL Protein-A Sepharose alone (VWR, Fontenay-sous-Bois, France), samples were incubated for 1 h at 4 °C with agitation in the presence of 0.5 µg of anti-ABCB4 (P3II-26 – Enzo Life Sciences, Villeurbanne, France) or unspecific IgG2b (MPC-11, BioLegend) antibodies, and then overnight at 4 °C with 40 µL Protein A-Sepharose. As a negative control allowing the elimination of unspecific contaminating proteins, we also included a condition with non-ABCB4-expressing HEK cells. Immunoprecipitates were recovered by centrifugation (1500 rpm, 5 min, 4 °C), washed three times with lysis buffer, and twice with ice-cold PBS. Bound proteins were eluted with 50 mM Tris-HCl pH 8.5 containing 2% SDS. As a control of immunoprecipitation efficacy, 30% of the eluted fraction was subjected to SDS-PAGE and analyzed by immunoblot with appropriate antibodies. The rest of the eluted samples was processed using the filtered-aided sample preparation (FASP) method to collect peptides as previously described [47]. Stage-tip-desalted peptides were analyzed by LC-MS-MS using an Ultimate 3000 Rapid Separation liquid chromatographic system coupled to an Orbitrap Fusion mass spectrometer (both from Thermo Fisher Scientific) as follows: peptides were loaded on a C18 reverse phase pre-column (3 μm particle size, 100 Å pore size, 75 μm inner diameter, 2 cm length; Thermo Fischer Scientific) using loading solvent (1% Acetonitrile and 0.1% trifluoroacetic acid in milliQ water) for 3 min at 5 µL.min^−1^, then separated on an analytical C18 reverse phase column (2 µm particle size, 100 Å pore size, 75 µm internal diameter, 25 cm length) with a 45 min effective gradient from 99% A (0.1% formic acid in milliQ water) to 50% B (80% Acetonitrile, 0.085% formic acid in milliQ water) at 400 nL.min^−1^. The mass spectrometer acquired data throughout the LC elution process and operated in a data-dependent scheme with full MS scans acquired with the orbitrap, followed by HCD fragmentation and ion trap fragment detection (top speed mode in 5 s) on the most abundant ions detected in the MS scan. Mass spectrometer settings were for full scan MS: AGC: 2.0E4, target resolution: 60,000, m/z range was 350–1500, maximum ion injection time: 60 ms; for HCD MS/MS: quadrupole filtering, normalized collision energy: 27. Ion trap rapid detection, intensity threshold: 1.0E4, isolation window: 1.6 m/z, dynamic exclusion time: 30 s, AGC Target: 2.0E4 and maximum injection time: 100 ms. The fragmentation was permitted for precursor with charge state of 2 to 7. Proteome discoverer 1.4 (Thermo Fisher Scientific) was used to generate. mgf peaklists files.

Peptides were identified as follows. Experimental mass lists were used to perform comparisons with theoretical mass lists from the *Homo sapiens* taxon of the Swiss-Prot database (May 2017, 20,204 sequences) using Mascot version 2.5.1 (www.matrixscience.com, accessed on 30 June 2021). The cleavage specificity set was the trypsin with maximum 2 missed cleavages. The precursor mass tolerance was set to 4 ppm and the MS/MS mass tolerance to 0.55 Da. Cystein carbamidomethylation was set as a constant modification while methionine oxidation was set as variable modification. With these settings, peptide identifications were considered as valid whenever their scores reached a minimum of 25, thus meeting the *p*-values criteria less than 0.05. The sample comparison was performed with MyPROMS software [48]. Identified proteins with at least 2 distinct peptides in at least one sample were considered positive.

### 4.4. Immunoblots, Immunofluorescence and Measurement of ABCB4-Mediated Phosphatidylcholine Secretion

Immunoblots were performed as previously described [9,45], using cells co-transfected with ABCB4- and RAB10-encoding constructs at a 1:1 ratio with the following primary antibodies: anti-ABCB4 (clone P3II-26) from Enzo Life Sciences, anti-α-tubulin (clone 1E4C11) from ProteinTech (Manchester, UK), anti-RAB10 (clone D36C4) from Cell Signaling (Danvers, MA, USA) and anti-GFP (clone AB10145) from Sigma. Immunoblots were quantified in the linear range of detection using ImageJ 1.50i software (U.S. National Institutes of Health, Bethesda, MD, USA).

For indirect immunofluorescence experiments, cells were grown on glass coverslips, fixed with 4% paraformaldehyde (Thermo Fisher Scientific) for 10 min, and permeabilized with 0.05% saponin in PBS containing 0.2% of bovine serum albumin (BSA). The coverslips were then incubated with the following primary antibodies: anti-ABCB4 and anti-RAB10 (see above), anti-calnexin (SPA-865, Enzo Life Sciences), anti-giantin (PRB-114C, Covance, Rueil-Malmaison, France), anti-EEA1 (sc-6415, Santa Cruz Biotechnology, Santa Cruz, CA, USA) and anti-LAMP1 (sc-20011, Santa Cruz Biotechnology); and subsequently incubated for 1 hour with appropriate Alexa Fluor-conjugated secondary antibodies (ThermoFisher Scientific). Nuclei were labeled using Hoechst 33342 (ThermoFisher Scientific). Immunofluorescence images were acquired using a confocal microscope (Eclipse TE-2000-Nikon-C2) equipped with a 60X objective, serial xy optical sections with a z-step of 0.3 μm were acquired using Nikon NIS-Elements software version AR 4.50 with constant settings (laser powers and correction of signal intensities) and treated using Adobe Photoshop version 8.0.1.

Alternatively, for specific cell surface labeling of ABCB4, non-permeabilized HeLa cells expressing ABCB4-FLAG were incubated 1 h with polyclonal anti-FLAG antibodies (F7425, Sigma) prior to fixation. Then, after fixation and permeabilization, total vs. cell surface ABCB4 were revealed using the standard procedure described above. A cell by cell quantification of cell surface vs. total ABCB4 signal intensities was performed as follows: individual ABCB4-expressing cells were randomly selected and delineated in ImageJ software, version 1.50i using the total ABCB4 signal. For each segmented cell, the fluorescence intensities associated with total ABCB4 (shown in blue) or plasma membrane ABCB4 (shown in red) were measured in each channel using the ‘Measure’ function of ImageJ. Then, after background subtraction (measured in areas with no apparent fluorescence) for each fluorescence intensity, plasma membrane over total ABCB4 fluorescence ratios were expressed as percentages of the mean ratio calculated for the reference condition (control).

The measurement of ABCB4-mediated PC secretion using a fluoro-enzymatic assay was performed as described [49] and results were analyzed as published [45].

### 4.5. Statistical Analyses

Graphics and one-way ANOVA tests were performed using Prism version 7.00 (GraphPad Software, La Jolla, CA, USA). A *p* value of less than 0.05 was considered significant with *: *p* < 0.05; **: *p* < 0.01; ***: *p* < 0.005; ns: not significant. Symbols always indicate the comparison between the control and the other tested conditions.

## Data Availability

The datasets generated and analyzed during the current study are available from the corresponding author on reasonable request.

## Supplementary Materials

The following are available online at https://www.mdpi.com/article/10.3390/ijms22137087/s1. Figure S1: Co-immunoprecipitation of ABCB4 and RAB10 in HEK cells. HEK cells co-expressing RAB10-GFP and ABCB4 were used. GFP (A) and ABCB4 (B) were immunoprecipitated using specific antibodies. Controls were performed using unspecific antibodies (unspe.). After SDS-PAGE, the presence of ABCB4 and RAB10-GFP in the lysates (Input) and the immunoprecipitates (IP) was detected by immunoblot (IB) as indicated. Molecular weight markers (in kDa) are shown. These panels are representative of three independent experiments per condition. Figure S2: Full immunoblots related to Figure 1A,B. Results shown in Figure 1A,B are delineated by dotted rectangles. MW (in kDa) are indicated. Figure S3: Phosphatidylcholine efflux after RAB10-WT expression. The phosphatidylcholine efflux from HEK cells expressing ABCB4-WT or RAB10-WT-GFP was measured as in Figure 2C. Note that for these experiments, means could not be normalized to ABCB4 expression levels. Means (± SD) of three independent experiments are shown. Figure S4: Full immunoblots related to Figure 2A. Results shown in Figure 2A are delineated by dotted rectangles. MW (in kDa) are indicated. Figure S5: ABCB4 immunolocalization in HepG2 cells. ABCB4 and the indicated forms of RAB10-GFP (GFP alone as control, ctrl) were co-expressed in HepG2 cells. Total ABCB4 and RAB10-GFP localization was analyzed as in Figure 3B. This figure is representative of three independent experiments. Bars: 10 µm. Figure S6: ABCB4-I541F-FLAG immunolocalization in HeLa cells. ABCB4-WT-FLAG or ABCB4-I541F-FLAG were expressed in HeLa cells. After treatment with vehicle (DMSO) or 10 µM cyclosporin A, cell surface and total ABCB4 were immunolabeled and visualized as in Figure 3B. This figure is representative of three independent experiments. Bars: 10 µm. Figure S7: Full immunoblots related to Figure 4A and Figure 5A. Results shown in Figure 4A and Figure 5A are delineated by dotted rectangles. MW (in kDa) are indicated. Figure S8: ABCB4 expression after RAB10 knockdown. Cell lysates from HeLa cells (A) and HEK cells (B) treated as in Figure 4 and Figure 5, respectively, were analyzed by immunoblot using the indicated antibodies (upper panels). MW (in kDa) are indicated. These panels are representative of three independent experiments per condition. ABCB4 signal intensities were quantified and means (± SD) of three independent experiments are shown (lower panels). Figure S9: Confocal microscopy analysis of RAB10-WT subcellular localization in HeLa cells. HeLa cells were transfected with a RAB10-WT-GFP-encoding construct (green) and the following intracellular compartments were immunolabeled (red): calnexin for endoplasmic reticulum, giantin for the Golgi apparatus, EAA1 for early endosomes, LAMP1 for late endosomes and lysosomes. This figure is representative of three independent experiments. Bars: 10 µm.

## Abbreviations

ABC: ATP-Binding Cassette; AP-MS: Affinity Purification-Mass Spectrometry; HEK: Human Embryonic Kidney; KO: KnockOut; PC: PhosphatidylCholine; RAB: RAs-related in Brain; siRNA: small interfering RNA; WT: Wild Type
