# Supplementary material for: RAB10 Interacts with ABCB4 and Regulates Its Intracellular Traffic
Source: Int J Mol Sci. 2021 Jun 30;22(13):7087. doi: 10.3390/ijms22137087 (PMC8268348; doi:10.3390/ijms22137087)
Supplement: Supplementary file 1 [file ijms-22-07087-s001.zip › ijms-1256849-supplementary.pdf]

RAB10 interacts with ABCB4 and regulates its intracellular traffic

Amel Ben Saad[1,2], Virginie Vauthier[2,3], Martine Lapalus[1], Elodie Mareux[1], Evangéline Bennana[4], Anne-Marie Durand-Schneider[2], Alix Bruneau[2,5], Jean-Louis Delaunay[2], Emmanuel Gonzales[1,6], Chantal Housset[2,7], Tounsia Aït-Slimane[2], François Guillonneau[4], Emmanuel Jacquemin[1,6], Thomas Falguières[1,*]

[1] Inserm, Université Paris-Saclay, Physiopathogénèse et traitement des maladies du foie, UMR_S 1193, Hepatinov, 91400 Orsay, France.

[2] Inserm, Sorbonne Université, Centre de Recherche Saint-Antoine (CRSA), UMR_S 938, Institute of Cardiometabolism and Nutrition (ICAN), 75012 Paris, France.

[3] Université de Paris, Institut Cochin, Inserm U1016, CNRS UMR 8104, 75014 Paris, France.

[4] 3P5-Proteom'IC platform, Université de Paris, Institut Cochin, Inserm U1016, CNRS UMR8104, 75014 Paris, France.

[5] Department of Hepatology & Gastroenterology, Charité Universitätsmedizin Berlin, 13353 Berlin, Germany.

[6] Assistance Publique - Hôpitaux de Paris, Paediatric Hepatology & Paediatric Liver Transplant Department, Reference Center for Rare Paediatric Liver Diseases, FILFOIE, ERN Rare-Liver, Faculté de Médecine Paris-Saclay, CHU Bicêtre, 94270 Le Kremlin-Bicêtre, France.

[7] Assistance Publique - Hôpitaux de Paris, Hôpital Saint-Antoine, Reference Center for inflammatory biliary diseases and autoimmune hepatitis, FILFOIE, ERN Rare-Liver, 75012 Paris, France.

[*] **Corresponding author:** Thomas Falguières, PhD – UMR_S 1193 Inserm / Université Paris Saclay – Rue des Adèles – Bâtiment 440 – F-91405 Orsay cedex, France. Phone: +33-(0)1-69-15-62-94. e-mail: thomas.falguieres@inserm.fr

Supplementary Table S1. Potential molecular partners of ABCB4 identified by AP-MS

	UniProt KB entry	MW	Proteins	Known fonction(s)
**MDR3_HUMAN**	**P21439**	**141.5**	**Phosphatidylcholine translocator ABCB4**	**PC efflux into bile from hepatocytes**
**RAB10_HUMAN**	**P61026**	**22.5**	**Ras-related protein Rab-10**	**Regulator of intracellular membrane trafficking, Golgi to PM traffic**
**RAB13_HUMAN**	**P51153**	**22.8**	**Ras-related protein Rab-13**	**Regulator of intracellular membrane trafficking**
**SEC23A_HUMAN**	**Q15436**	**86.2**	**Protein transport protein Sec23A**	**COPII component, ER-to-Golgi transport**
**SKP1_HUMAN**	**P63208**	**18.7**	**S-phase kinase-associated protein 1**	**Essential component of the SCF ubiquitin ligase complex**
**TXLNG_HUMAN**	**Q9NUQ3**	**60.6**	**Gamma-taxilin**	**Involved in intracellular vesicle traffic-binds to syntaxin family members 1A, 3A and 4A**
**DAD1_HUMAN**	**P61803**	**12.5**	**Dolichyl-diphosphooligosaccharide**	**Transfer of mannose oligosaccharide to nascent polypeptide**
**RHEB_HUMAN**	**Q15382**	**20.5**	**GTP-binding protein Rheb**	**Activates the protein kinase activity of mTOR**
**SKP1_HUMAN**	**Q9NP81**	**18.7**	**S-phase kinase-associated protein 1**	**Component of the SCF ubiquitin ligase complex**
**UCHL1_HUMAN**	**P09936**	**24.8**	**Ubiquitin carboxyl-terminal hydrolase isozyme L1**	**Processing of ubiquitin precursors and of ubiquitinated proteins**
**DNJC8_HUMAN**	**O75937**	**29.8**	**DnaJ homolog subfamily C member 8**	**DnaJ heat shock protein family (Hsp40)**
ODPA_HUMAN	P08559	43.3	Pyruvate dehydrogenase E1 component subunit alpha	Catalyzes the overall conversion of pyruvate to acetyl-CoA and CO_2
DHRS3_HUMAN	O75911	33.5	Short-chain dehydrogenase/reductase 3	Catalyzes the reduction of all-trans-retinal to all-trans retinol
RAI3_HUMAN	Q8NFJ5	40.3	Retinoic acid-induced protein 3	Protein involved in interaction between retinoic acid and G proteins
FMR1_HUMAN	Q06787	71.2	Synaptic functional regulator FMR1	Multifunctional polyribosome-associated RNA-binding protein
FIP1_HUMAN	Q6UN15	66.5	Pre-mRNA 3'-end-processing factor FIP1	Contributes to poly(A) site recognition and stimulates poly(A) addition
MCES_HUMAN	O43148	54.8	mRNA cap guanine-N7 methyltransferase	mRNA-capping methyltransferase
NOG2_HUMAN	Q13823	83.7	Nucleolar GTP-binding protein 2	GTPase that associates with pre-60S ribosomal subunits in the nucleolus

ABCB4 and RAB10 are highlighted. Proteins of potential interest are shown in bold.

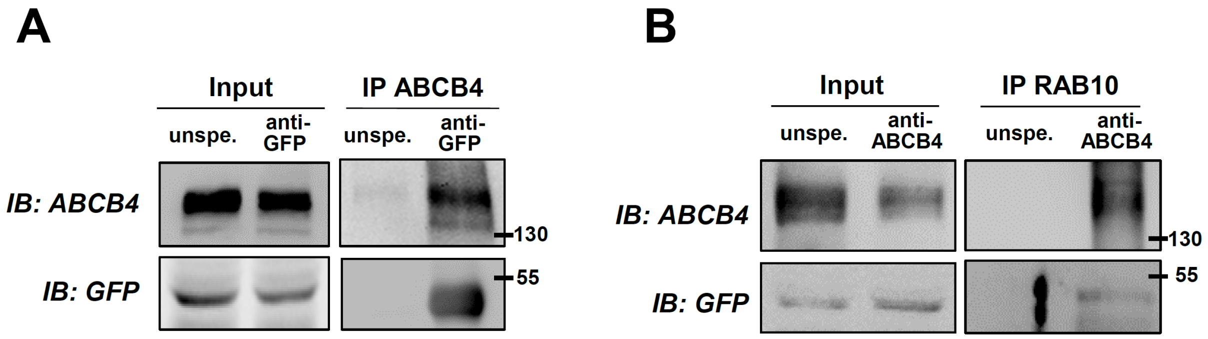

Figure S1. Co-immunoprecipitation of ABCB4 and RAB10 in HEK cells. HEK cells co-expressing RAB10-GFP and ABCB4 were used. GFP (A) and ABCB4 (B) were immunoprecipitated using specific antibodies. Controls were performed using unspecific antibodies (unspe.). After SDS-PAGE, the presence of ABCB4 and RAB10-GFP in the lysates (Input) and the immunoprecipitates (IP) was detected by immunoblot (IB) as indicated. Molecular weight markers (in kDa) are shown. These panels are representative of three independent experiments per condition.

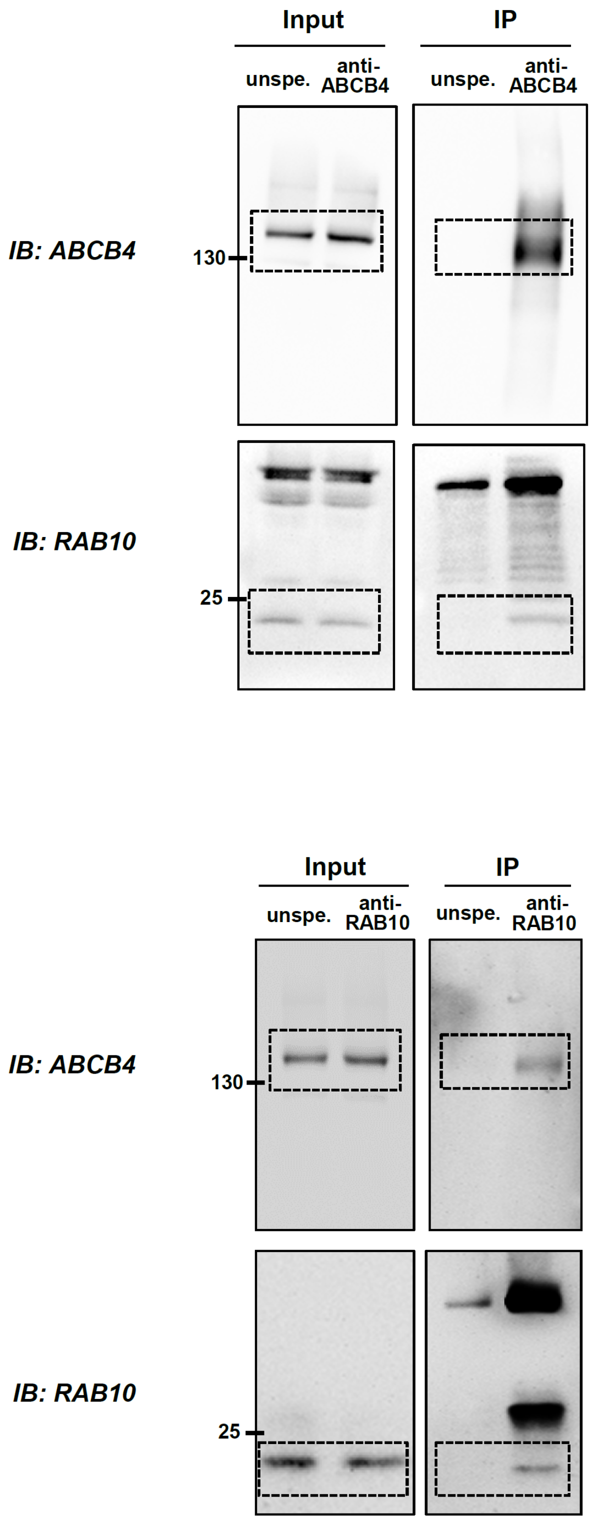

Figure S2. Full immunoblots related to Figure 1A-B. Results shown in Figure 1A-B are delineated by dotted rectangles. MW (in kDa) are indicated.

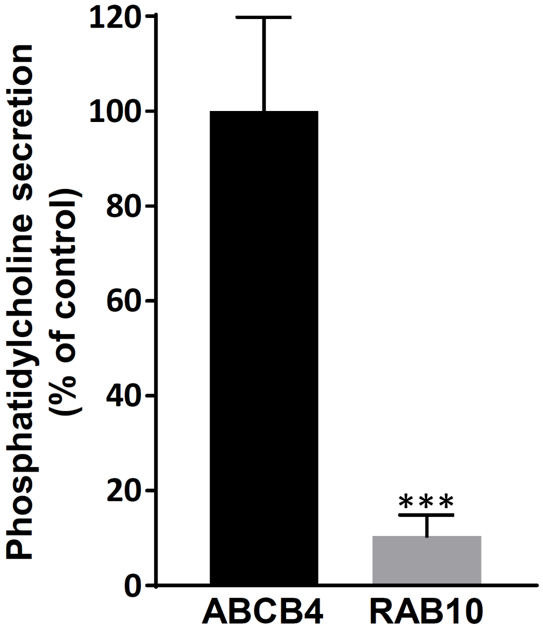

Figure S3. Phosphatidylcholine efflux after RAB10-WT expression. The phosphatidylcholine efflux from HEK cells expressing ABCB4-WT or RAB10-WT-GFP was measured as in Figure 2C. Note that for these experiments, means could not be normalized to ABCB4 expression levels. Means (± SD) of three independent experiments are shown.

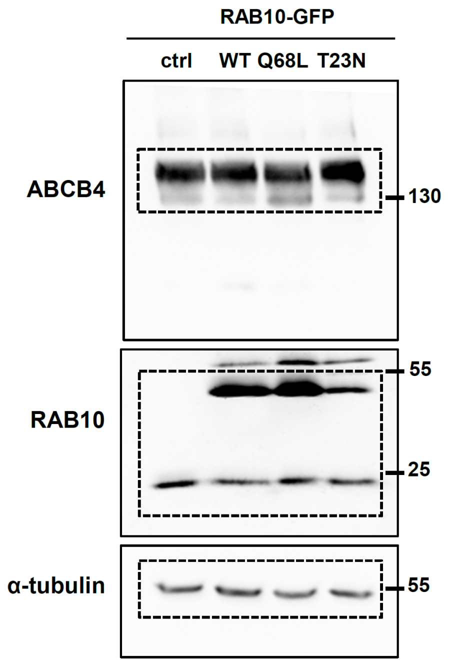

Figure S4. Full immunoblots related to Figure 2A. Results shown in Figure 2A are delineated by dotted rectangles. MW (in kDa) are indicated.

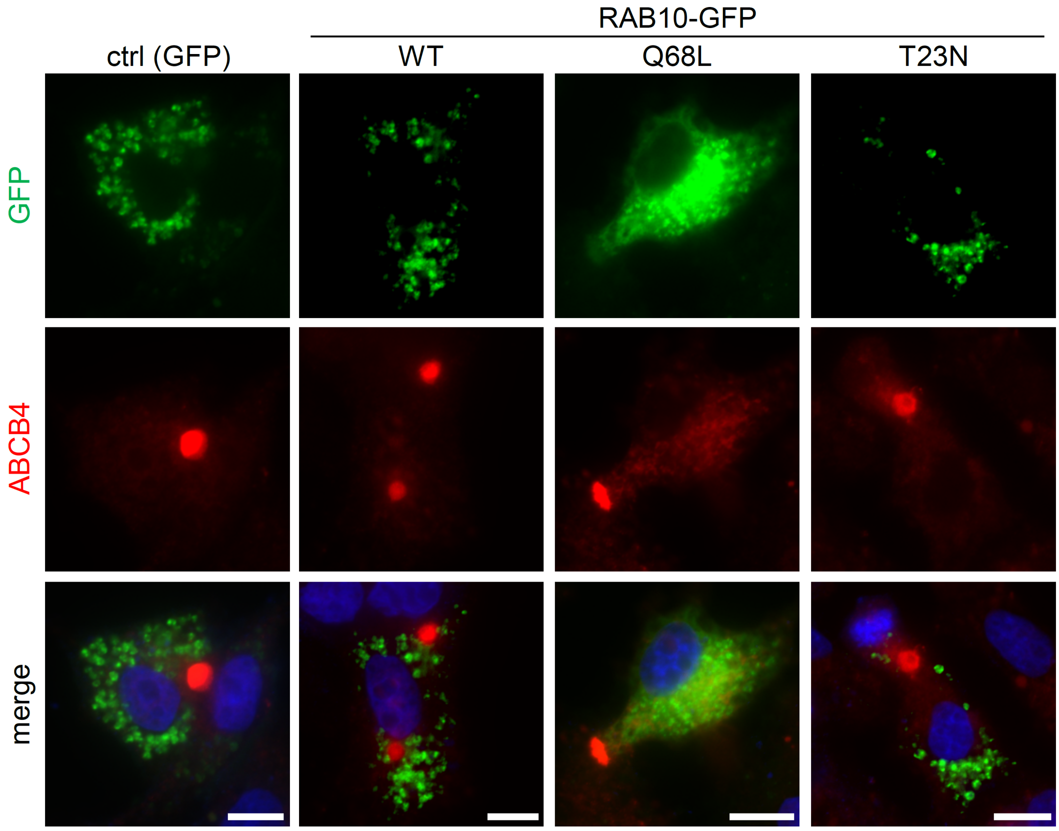

Figure S5. ABCB4 immunolocalization in HepG2 cells. ABCB4 and the indicated forms of RAB10-GFP (GFP alone as control, ctrl) were co-expressed in HepG2 cells. Total ABCB4 and RAB10-GFP localization was analyzed as in Figure 3B. This figure is representative of three independent experiments. Bars: 10 µm.

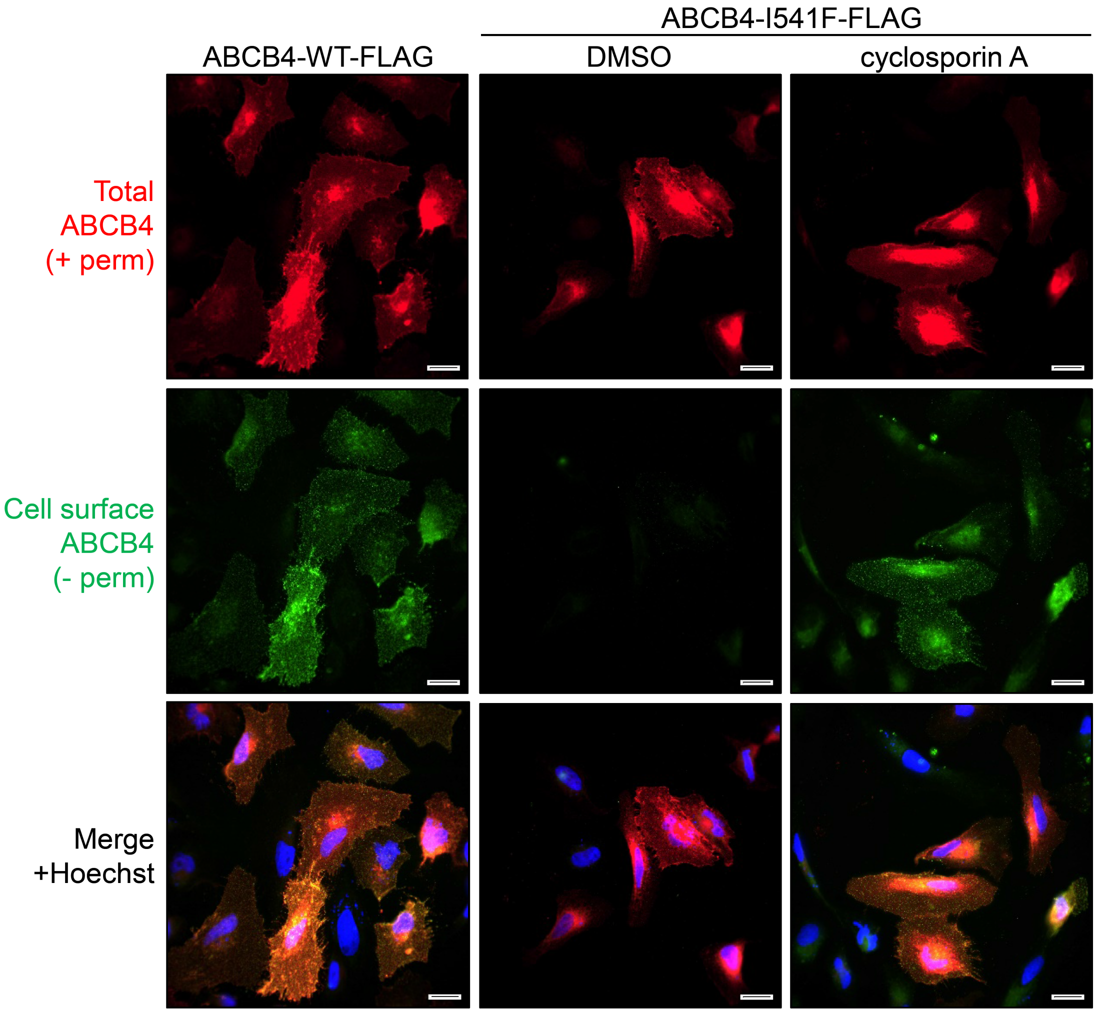

Figure S6. ABCB4-I541F-FLAG immunolocalization in HeLa cells. ABCB4-WT-FLAG or ABCB4-I541F-FLAG were expressed in HeLa cells. After treatment with vehicle (DMSO) or 10 µM cyclosporin A, cell surface and total ABCB4 were immunolabeled and visualized as in Figure 3B. This figure is representative of three independent experiments. Bars: 10 µm.

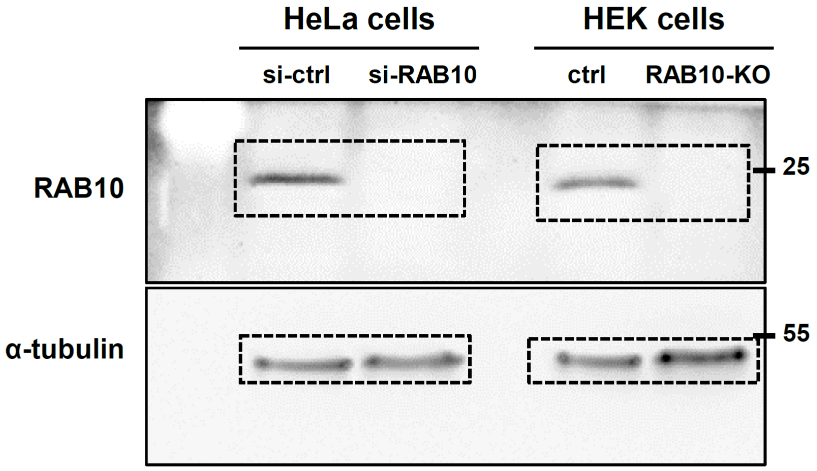

Figure S7. Full immunoblots related to Figures 4A and 5A. Results shown in Figures 4A and 5A are delineated by dotted rectangles. MW (in kDa) are indicated.

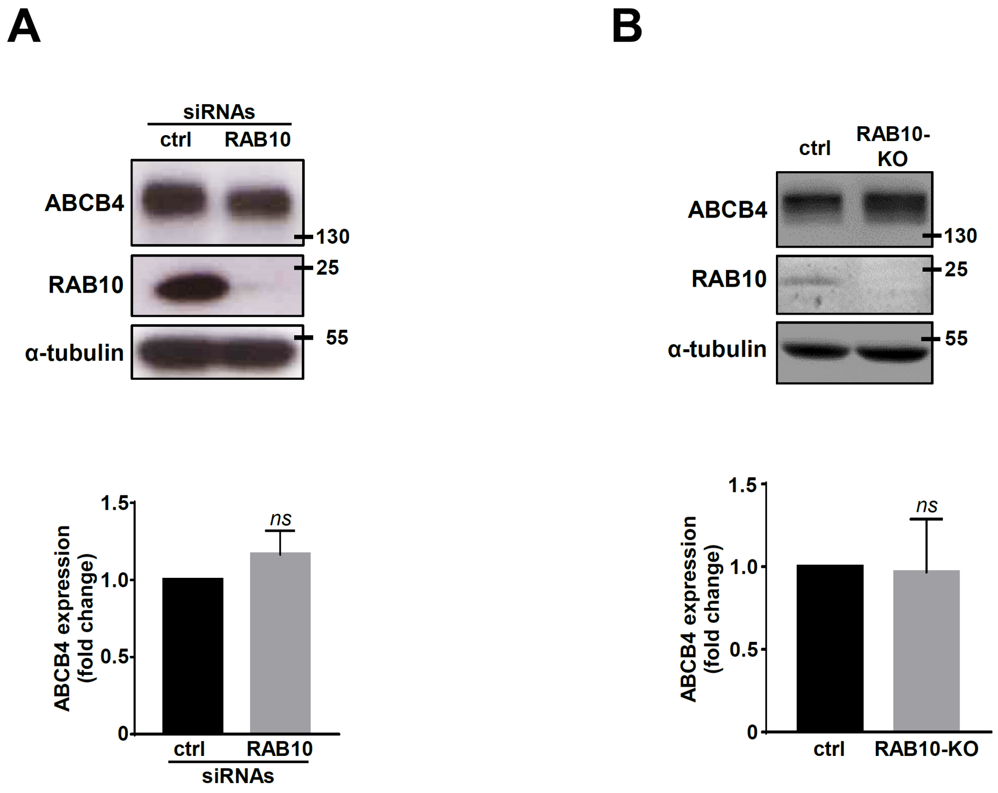

Figure S8. ABCB4 expression after RAB10 knockdown. Cell lysates from HeLa cells (A) and HEK cells (B) treated as in Figures 4 and 5, respectively, were analyzed by immunoblot using the indicated antibodies (upper panels). MW (in kDa) are indicated. These panels are representative of three independent experiments per condition. ABCB4 signal intensities were quantified and means (± SD) of three independent experiments are shown (lower panels).

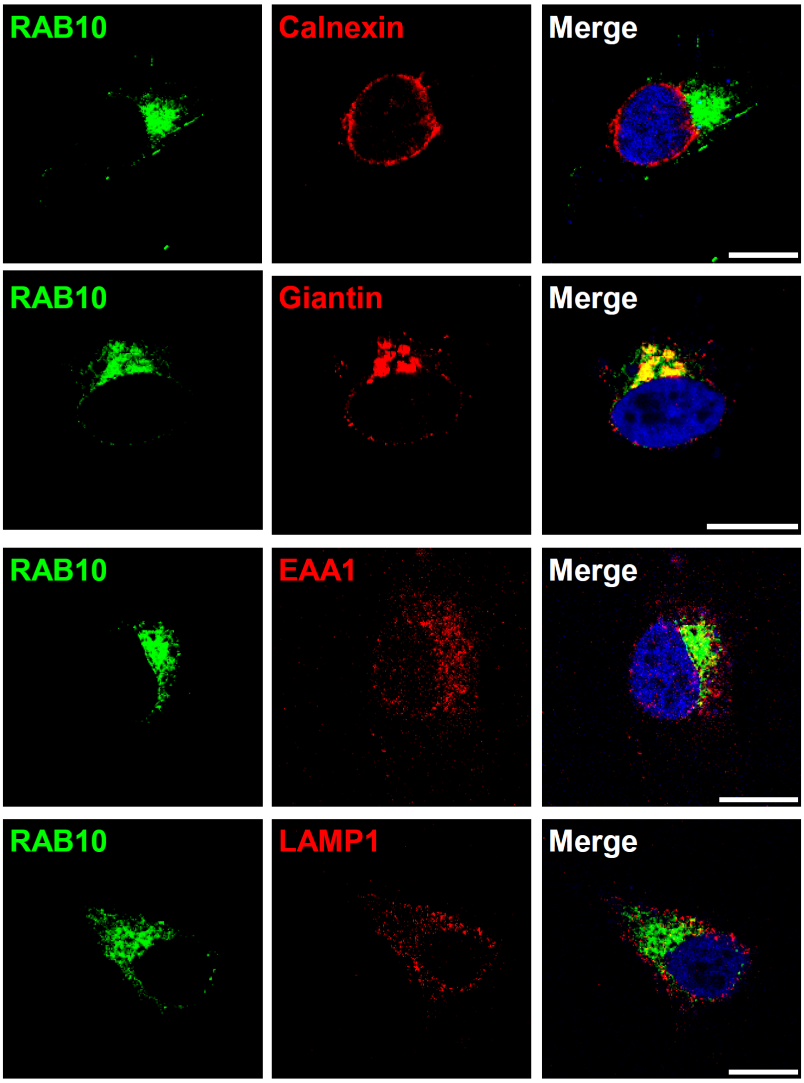

Figure S9. Confocal microscopy analysis of RAB10-WT subcellular localization in HeLa cells. HeLa cells were transfected with a RAB10-WT-GFP-encoding construct (green) and the following intracellular compartments were immunolabeled (red): calnexin for endoplasmic reticulum, giantin for the Golgi apparatus, EAA1 for early endosomes, LAMP1 for late endosomes and lysosomes. This figure is representative of three independent experiments. Bars: 10 µm.
